# Lipophilic Metabolites and Anatomical Acclimatization of *Cleome amblyocarpa* in the Drought and Extra-Water Areas of the Arid Desert of UAE

**DOI:** 10.3390/plants8050132

**Published:** 2019-05-16

**Authors:** Sameh S.M. Soliman, Mohamed Abouleish, Maged M.M. Abou-Hashem, Alshaimaa M. Hamoda, Ali A. El-Keblawy

**Affiliations:** 1 Sharjah Institute for Medical Research and College of Pharmacy, University of Sharjah, P.O. Box 27272, Sharjah, UAE; alshaimaahamoda@gmail.com; 2 Department of Pharmacognosy, Faculty of Pharmacy, Zagazig University, Zagazig 44519, Egypt; maged_a_hashem@hotmail.com; 3 Department of Biology, Chemistry and Environmental Sciences, College of Arts and Sciences, American University of Sharjah, P.O. Box 26666, Sharjah, UAE; mabouleish@aus.edu; 4 Department of Plant Biology, University of Malaga, 29071 Malaga, Spain; 5 Department of Pharmacognosy, Faculty of Pharmacy, Assiut University, Assiut 71526, Egypt; 6 Department of Applied Biology, University of Sharjah, P.O. Box 27272, Sharjah, UAE; akeblawy@sharjah.ac.ae; 7 Research Institutes of Science and Engineering, University of Sharjah, P.O. Box 27272, Sharjah, UAE

**Keywords:** arid desert of United Arab Emirates, drought stress, rare plant, *Cleome amblyocarpa*, lipid metabolites, shaggy-like trichomes

## Abstract

Plants adapt to different environmental conditions by developing structural and metabolic mechanisms. In this study, anatomical features and lipophilic metabolites were investigated in *Cleome amblyocarpa* Barr. & Murb., *Cleomaceae* plants growing in the arid desert of United Arab Emirates (UAE) in either low-water or extra-water areas, which were caused by the surrounding road run-off. The plant showed the presence of shaggy-like trichomes. The plant also developed special mechanisms to ensure its survival via release of lipophilic metabolites. The lipophilic metabolites, stained red with Sudan III, were apparently released by glandular trichomes and idioblasts of the shoot and roots, respectively. The identified lipophilic metabolites included those required for drought tolerance, protection against pathogens invasion, and detoxification. Plants growing in the low-water area caused an increase in the production of lipophilic metabolites—in particular, hydrocarbons and terpenoids. The lipophilic metabolites are known to provide the plant with unique waxy surfaces that reduce water loss and avoid penetration by pathogens. The release of lipid metabolites and the presence of shaggy-like trichomes represented unique features of the species that have never been reported. The provided chemical ecology information can be extended for several plant-related applications, particularly including drought tolerance.

## 1. Introduction

Individual plants perceive an enormous range of external cues. In the hyper-arid Arabian Desert, plants face several types of stresses, such as drought and high temperatures. Plants respond and adapt to stress conditions through complex regulatory networks, which are better understood as of late due to the application of genomics, transcriptomics, proteomics, and metabolomics analyses [1]. Under drought stress, plants induce different biochemical and physiological activities that affect plant growth, development, and metabolism [2]. It is reported that drought stress affects the production of several metabolites. For example, Bouaziz et al. (2009) reported that plants of the arid deserts of south Tunisia developed high resistance to several abiotic stresses, such as drought, saltiness, nitrogen limitation, and light exposure, through the production of a high content of natural antioxidants, such as phenolic compounds [3].

Environmental conditions including drought stress affect plant growth and metabolism [4]. Drought interferes with the plant development and weakens its defense; consequently, the plants become more susceptible to infection with pathogens [5]. For example, during the flowering stages, hot and dry conditions increase the populations of pathogenic fungus *Aspergillus flavus* [6]. In the natural environment, plants have evolved an elaborate, multi-layered protection system, including the plant cell wall and the plasma membrane, which prevent pathogen penetration and account for the majority of aborted infections [7].

It has been reported that the production of reactive oxygen species (ROS) in plant cells is low under normal growth conditions [8]. However, many stresses, such as drought stress and desiccation, disrupt the cellular homeostasis and enhance the production of ROS [8]. Antioxidants such as ascorbic acid and glutathione are crucial for plant defense against oxidative stress of ROS [9]. Furthermore, the cellular damage caused by these radicals is mediated by interactions with different cellular constituents, including lipids. 

Lipids and lipophilic metabolites are functioning as secondary signal molecules and also have a role in mediating cross talk between signaling mechanisms in plant defense against biotic stress [10,11]. Additionally, these metabolites provide a physical barrier on the surface of epidermal cells that protects the plant from environmental assaults [12].

Twelve *Cleome* species have been recorded in the flora of the United Arab Emirates (UAE). These are *Cleome amblyocarpa*, *Cleome arabica*, *Cleome austroarabica*, *Cleome brachycarpa*, *Cleome dolichostyla*, *Cleome droserifolia*, *Cleome gynandra*, *Cleome noeana*, *Cleome quinquenervia*, *Cleome rupicola*, *Cleome scaposa*, and *Cleome viscose* [13,14]. *Cleome*, known as spider flowers, is a genus of flowering plants in the *Cleomaceae* [15]. *C. amblyocarpa* Barr. & Murb. is an annual herb abundant in sandy environments as well as the gravel and stony grounds in the arid Arabian deserts. The plant height can reach more than 50 cm in a rainy year with rigid, erect, and branched stems and alternate trifoliate leaves. The plant growing in the UAE tolerates the unique environmental conditions, particularly including drought. However, the mechanisms that provide these tolerances have never been investigated. 

**Figure 1:**
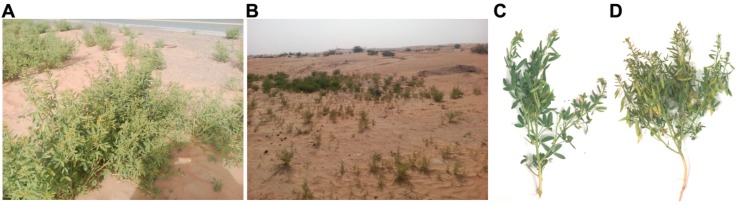
*Cleome amblyocarpa* plant in the Arabian arid desert of the United Arab Emirates (UAE). (**A**) Plants growing in an extra-water area caused by a surrounding road run-off. (**B**) Plants growing in a low-water area. (**C**) Individual plant that received extra-water growing in a very dry year. (**D**) Individual plant growing in dry sandy soil in a very dry year.

Most phytochemical screening of *C. amblyocarpa* has been done for medical purposes [16,17]. However, no study has assessed the effect of drought on the plant metabolism. Furthermore, there has been no attempt to investigate the role of lipophilic metabolites produced by this species in an ecological context. This study provides insight into how the plant evolves metabolic mechanisms to acclimatize to stressful conditions, including dry sandy soil in a very dry year, as compared to plants growing in an over-watered area caused by a surrounding road run-off (Figure 1). The study also categorizes the identified lipophilic metabolites based on their ecological functions to those associated with drought tolerance, pathogen protection, and survival. Lipid metabolites include fatty acids (FA) as well as their reduced forms, including aldehydes and alcohols [18]. Lipid metabolites also can include hydrocarbons (HC) that are biosynthesized either from FA or terpenoids by a head-to-head manner [18]. Furthermore, terpenoids are major contributors in the lipid metabolism and accumulation [19]. 

## 2. Material and Methods

### 2.1. Plant Material

Fresh mature plants of *C. amblyocarpa* were collected from the Faya region near Sharjah, UAE. The plant was taxonomically identified by Professor Ali El-Keblawy from the department of Applied Biology, University of Sharjah, UAE, and voucher specimens were deposited at the University Herbarium. Plants were collected on a rainy day from two very close sites on March 2018. One site represented an extra-water area caused by a surrounding road run-off (Figure 1A). However, the other site received the normal rainfall, which was very low during the year of 2018 (7 times with an average 5 mm between September to May) (Figure 1B). According to the nearest meteorological station (coordinates 25.3284° N, 55.5123° E) close to the study site, the total amount of rainfall received in the study year was less than 40 mm according to the Online World Weather, compared to 103 mm for a long term average [20]. Figure 1 shows the difference in growth between plants growing at the over-watering area (Figure 1C) caused by a surrounding road run-off and those growing at the low-watering area (Figure 1D). 

### 2.2. Lipophilic Metabolites Extraction

The plant (~10 plants from each site) was dissected into leaves, fruits, stems, and roots. Each plant organ representing ~200 gm was extracted separately by direct steam distillation according to [21]. The collected turbid water distillate was extracted with chloroform. The chloroform layer was cleaned by anhydrous sodium sulphate and then analyzed by gas chromatography-mass spectrometry (GC-MS). Three different samples were used for lipophilic metabolites extraction. 

### 2.3. GC-MS of C. amblyocarpa Plants Collected from Both Sites

The analyses were carried out using an Agilent Model 7683 Autosampler, 6890 Gas Chromatograph, and 5975 Inert Mass Selective Detector in the electron impact (EI) mode according to Soliman et al., 2018 [22]. Data collection and analysis were performed using MSD Enhanced Chemstation software (Agilent). Product spectra were identified by comparison of the measured fragmentation patterns to those found in the NIST 08 Mass Spectral Library. 

### 2.4. Light Microscopy

Young and fully-grown leaves, mature fruits, stems (median portion), and roots (lower half) from 4–8 adult individuals from each population were examined, and their anatomy was described. The samples were subjected to very thin, hand-made sectioning followed by either fixation directly on microscopic slides or stained with Sudan III. The sections were stained with 1% Sudan III for 5 min, followed by washing with 99% ethanol three times prior to fixation and examination by light microscopy. The plant sections were observed under light microscopy (Optika, B-290TB, Italy) using both magnification powers 10 and 40 [22]. The number of trichomes on leaves, stems, and fruits per plant (3–5 plants per population) was counted per at least 10 fields, and the average was calculated. Student *T*-test was used to measure the significance differences. *P*-value <0.05 was considered significant. 

## 3. Results and Discussion

Plants develop special mechanisms in order to tolerate their environmental conditions. *C. amblyocarpa* is an annual plant growing in the Middle East, including in UAE. UAE is known for its arid environmental conditions. The plant growing in the UAE developed anatomical and metabolic mechanisms in order to tolerate the arid climate conditions. However, the level and the type of metabolites may vary in response to the amount of water received per year. In this study, we compared *C. amblyocarpa* plants growing under the drought conditions of UAE either in low-water areas or extra-water areas caused by a surrounding road run-off.

### 3.1. Anatomical Characteristics of C. amblyocarpa in the Arid Dessert of UAE

**Figure 2:**
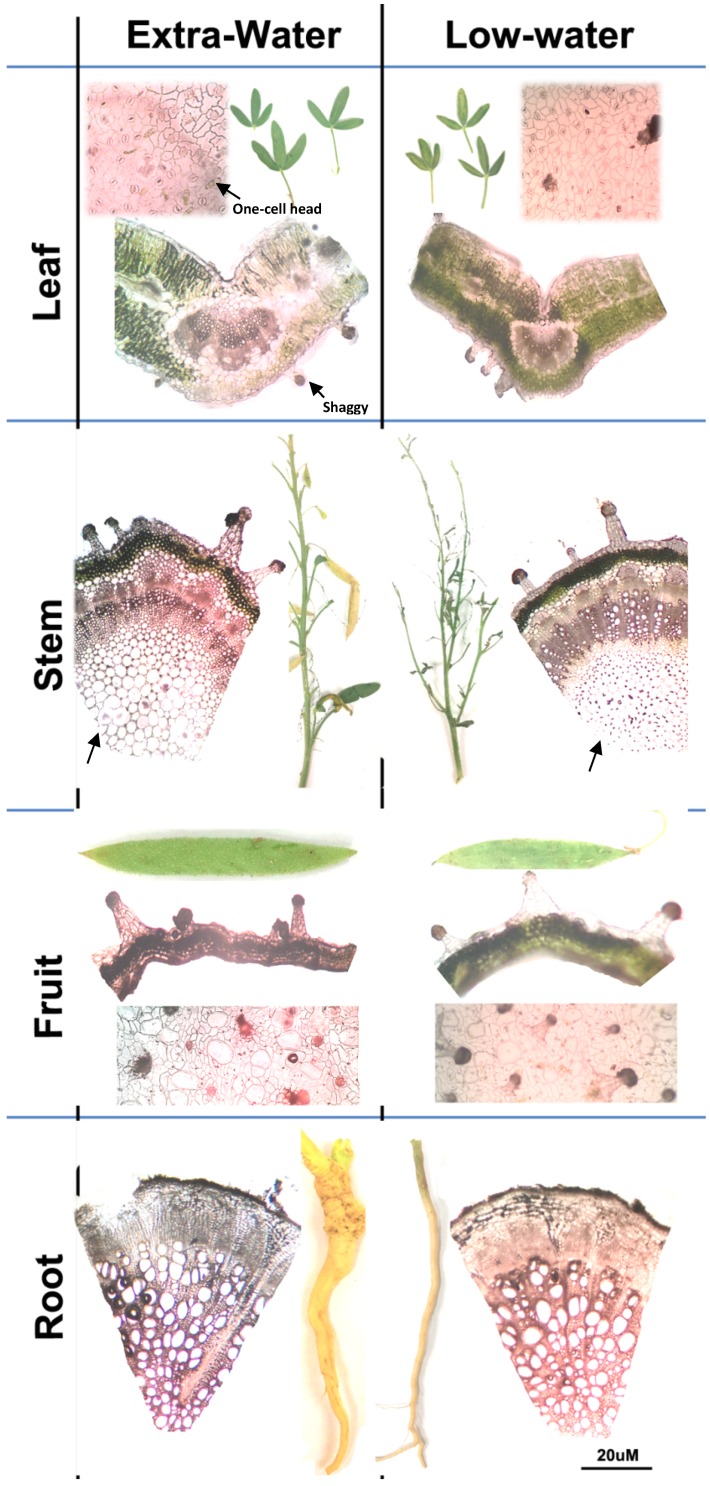
Comparative anatomical study of leaves, stems, fruits, and roots of the *C. amblyocarpa* plant growing in the Arabian arid desert of UAE at two areas—one receiving extra-water and one receiving low-water. Arrows indicate types of trichomes and intercellular spaces.

*C. amblyocarpa* growing in the UAE showed numerous glandular trichomes of multiseriate-multicellular stalks and multicellular heads similar to shaggy trichomes [23]. The trichomes were distributed throughout the plant leaves, stems, and fruits (Figure 2). There were no significant differences (Student-T test, *P-*value = 0.19) in the number of trichomes between plants growing in the low-water area (7.6 ± 3.8) and those growing in the extra-water area (9.3 ± 1.2) (Figure 2). Furthermore, few one-cell head glandular trichomes were noticed on the leaf surface (Figure 2). The glandular trichomes of *C. amblyocarpa* were previously reported but have never been described [24]. Several types of trichomes have been linked to the increase in water use efficiency and to reflect broad-spectrum electromagnetic radiation, reducing light absorbance and modulating energy balance [25]. Plant trichomes may also provide the plant with extra barriers to protect the plant from pathogen invasion and water loss [26] by secreting various metabolites, including lipophilic metabolites [27]. 

The UAE *C. amblyocarpa* showed compact mesophyll with no intercellular spaces and wide vascular bundles (Figure 2). This is in accordance to previously-reported data for plants exposed to drought conditions [28,29]. Absence of intercellular spaces in the mesophyll layer is an indication of resistance to water flow in plants. For example, drought-stressed wheat showed a compact mesophyll layer without intercellular spaces and with an increase in the width of xylem vessels for the purpose of an efficient water absorption [28]. 

**Figure 3:**
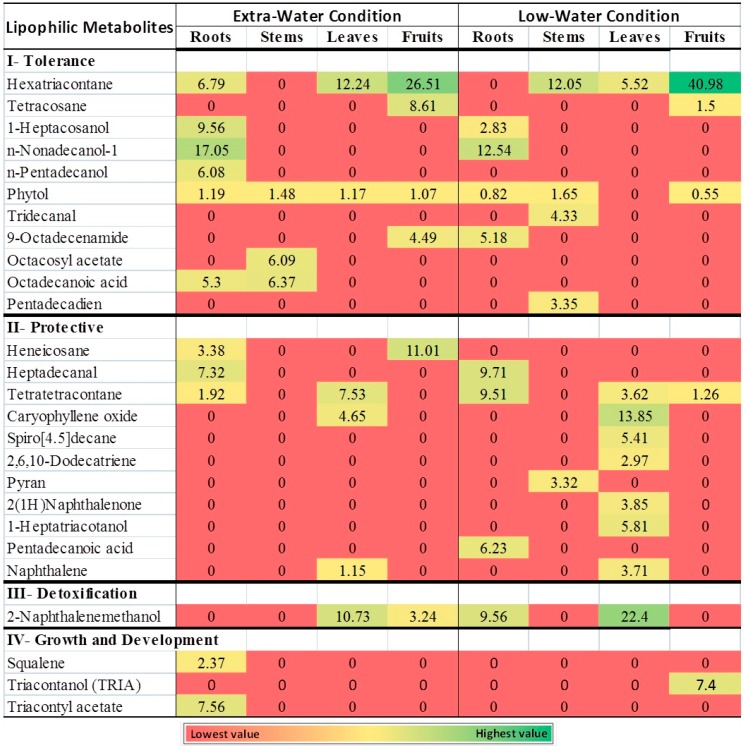
Heatmap comparing the gas chromatography-mass spectrometry (GC-MS) analysis of the *C. amblyocarpa* plant growing either in extra-water or low-water conditions of the arid desert of Sharjah, UAE. The metabolite averages and relative percentages of three replicates are displayed as colors ranging from red to green, as shown above. The relative percentage of a metabolite is represented in relation to total areas of all detected metabolites in an extract.

Compared to plants that received extra-water (Figure 1C), it was observed that *C. amblyocarpa* that received low-water (Figure 1D) was characterized by more pale colored leaves and fruits, thinner, dull roots, and numerous smaller fruits (Figure 2). The pale color of the plants may be attributed to the production of naphthalenone (Figure 3). Naphthalenone is known to have tyrosinase inhibitory activity [30] that may cause plant de-pigmentation. 

### 3.2. C. amblyocarpa Growing at the Arid Dessert of UAE Produced Lipophilic Metabolites 

#### 3.2.1. Lipophilic Metabolites and Drought Tolerance 

The *Cleome* genus is known as a source of several metabolites, including essential oils, terpenes, and glucosinolates [31]. The analysis of lipophilic metabolites of UAE *C. amblyocarpa* revealed the production of lipophilic substances by the plant, such as hexatriacontane [32] (detected in roots, leaves, and fruits), tetracosane [33] (found in the plant fruits), and 1-heptacosanol (detected in the roots). Phytol was also detected in all plant organs except the leaves. The presence of phytol indicated its important role in tolerance to high temperatures and long exposures to light [34]. Additionally, n-nonadecanol-1, n-pentadecanol, octacosyl acetate, octadecanoic acid, and hexatriacontane are all lipid metabolites that were detected in *C. amblyocarpa*. These metabolites are known to play an important role in the drought tolerance of plants [32] (Figure 3, Supplementary Figure S1 and Supplementary Table S1). 

Plants growing in the low-water area showed abundant levels of the aforementioned metabolites, including hexatriacontane (disappeared from the roots), tetracosane, 1-heptacosanol, and tridecanal [35], which was found in plant stems (Figure 3 and Supplementary Table S1). Furthermore, a lipid metabolite, 9-octadecenamide (Z) (oleamide), known to play an important role in making the plant surfaces slide [36,37], was found only in the roots of plants growing in the low-water area (Figure 3 and Supplementary Table S1). 

#### 3.2.2. Lipophilic Metabolites and Protection from Pathogens Invasion 

The plant can avoid pathogen invasion and herbivore attack in the arid desert of UAE by producing lipophilic metabolites known for antimicrobial, antifungal, insect repellent, and cytotoxic activities. The antimicrobial metabolite, heneicosane [38,39], was detected in roots and fruits of the plant. The antimicrobial and cytotoxic metabolite, caryophyllene oxide [40], was accumulated in the plant leaves, while heptadecanal [41] was accumulated in the plant roots. Moreover, the antifungal, tetratetracontane [42], was found accumulated in the plant roots and leaves (Figure 3). 

Protective lipophilic metabolites produced by the plants growing in the low-water area included caryophyllene oxide accumulated in the plant leaves, heptadecanal accumulated in the roots, and tetratetracontane accumulated in the roots, leaves, and fruits (Figure 3 and Supplementary Table S1). Other lipid metabolites produced by the plants growing in the low-water area included: spiro[4.5]decane, a plant defensive metabolite [43,44] found accumulated only in the leaves; pyran, which plays an important role in plant protection [45] and was found only in the stem; 2(1H)naphthalenone [46,47] was abundant in the leaves and fruits; and heptatriacotanol [48,49] was accumulated in the plant leaves (Figure 3 and Supplementary Table S1). Additional protective lipid metabolites included heptadecanal, pentadecanoic acid, and tetratetracontane (Figure 3 and Supplementary Table S1). 

#### 3.2.3. Lipophilic Metabolites and Detoxification 

The plant also tolerated the rise of toxins due to drought and desiccation conditions [8] by producing xenobiotic metabolites, such as 2-naphthalene methanol [50]. Naphthalene methanol was detected in the aerial parts of the plant, including the leaves and fruits (Figure 3 and Supplementary Table S1). Naphthalene methanol was not detected when another extract (employed as a control) was injected similarly in the GC-MS, indicating that the metabolite was a real plant product. The plants from the low-water area showed double the production of 2-naphthalenemethanol when compared to the plants growing in the area receiving extra-water (Figure 3 and Supplementary Table S1). 

#### 3.2.4. Lipophilic Metabolites and Growth And Development

Squalene and triacontanol (TRIA) were the major metabolites detected in the *C. amblyocarpa* plant growing in UAE. Both metabolites are known for their importance in plant growth and development. Squalene plays an important role in steroid biosynthesis, which is an essential component in the structure of plant cell walls [51]. TRIA is a potent plant growth regulator found in epicuticular waxes. TRIA is a fatty alcohol known to improve growth, photosynthesis, protein synthesis, uptake of water and nutrients, nitrogen-fixation, and enzymes activities of many plants [52]. It has been reported that plants can reduce the negative effects of drought conditions through the production of dense trichomes and thick cuticular waxes on the plant leaves [53]. 

### 3.3. Lipophilic Metabolites Localization: Roots versus Aerial Parts 

Compared to plants receiving extra-water, the roots of the plants growing at the low-water condition showed the appearance of new compounds, such as 9-octadecenamide (Z) and pentadecanoic acid. Additionally, the roots showed the disappearance of hexatriacontane, pentadecanol (an indicator of oxidation during stress), tetratriacontane, and triacontyl acetate (Figure 3, Supplementary Figure S1, and Supplementary Table S1). 

In comparison to the plants growing in the extra-water condition, the stems of the plants growing in the low-water condition showed the disappearance of octacosyl acetate and octadecanoic acid, the over-production of tridecanal, and the appearance of pentadecadien and hexatriacontane. The reported metabolites are known to provide super hydrophobic plant surfaces [54], which may help the plant to avoid water loss during heat stress and long days [55] (Figure 3, Supplementary Figure S1, and Supplementary Table S1). Similarly, leaves showed lower levels of tetratetracontane and hexatriacontane, the disappearance of 2,6-dihexadecanoate and phytol, and the over-production of detoxifying metabolite 2-naphthalenemethanol as well as tolerance metabolites such as hexatriacontane, caryophyllene oxide, and naphthalene. On the other hand, fruits showed the appearance of 2(1H)naphthalenone, tetratetracontane and triacontanol (TRIA) and the disappearance of 2-naphthalenemethanol (an indicator of photo-cleavage [56]), 9-octadecenamide, and heneicosane, indicating down-production under the low-water condition [57]. It is clearly indicated that more stress due to low-water conditions increased the production of the protective metabolites in the leaves as well as the metabolites required for tolerance in the roots. 

**Figure 4:**
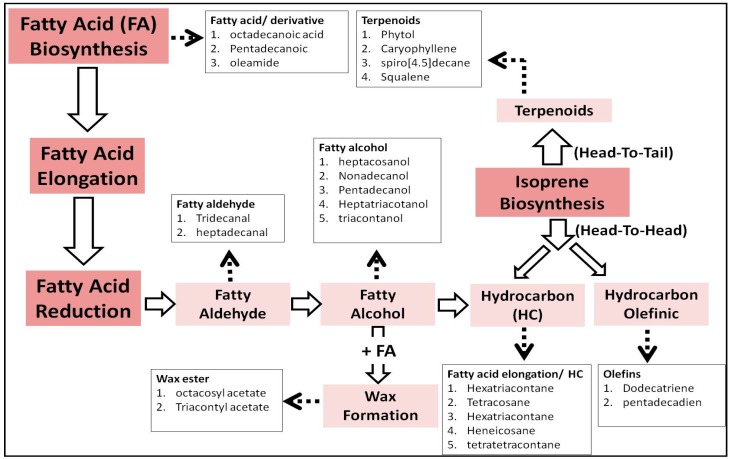
Schematic summary of the biosynthesis and the metabolism of lipid metabolites described in the study [18].

From the biosynthetic point of view, lipid metabolites are biosynthesized either from fatty acids or terpenoids (Figure 4). Fatty acid accumulation may be associated with the reduction process for the formation of aldehyde, alcohols, or hydrocarbons, or with esterification for the formation of wax [18] (Figure 4). FAs were more abundant in the stems; wax and FA-ester were abundant in the stems and roots, while the reduced forms of FA were more accumulated in the roots and the aerial parts. FA alcohols were abundant in the roots, and HCs were abundant in the leaves and fruits. Similar observations were obtained for plants growing at the low-water condition, while the FA and the wax disappeared from the stems, and more HCs were accumulated in the aerial parts. The protective lipid metabolites were accumulated as reduced forms of FA (HC and aldehyde) in the roots and the leaves of the plants growing at the extra-water condition. The terpenoids (including phytol) were accumulated in the aerial parts and the roots of both extra- and low-water conditions. Other terpenoids, including caryophyllene derivative, spiro-decane, and olefins, were more abundant in the leaves of plants growing at the low-water condition. The results obtained indicated that environmental factors—particularly drought stress—can affect the plant lipids accumulation and composition [58]. 

**Figure 5:**
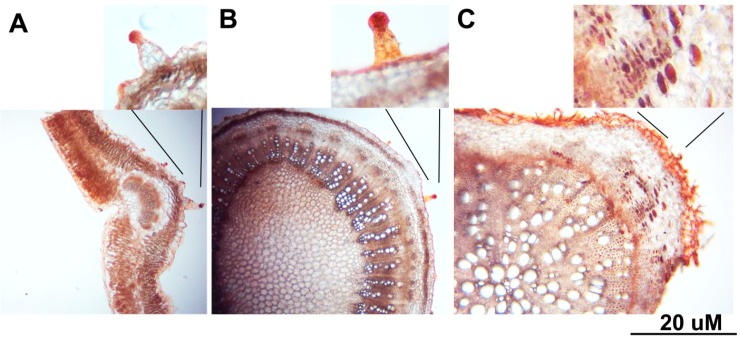
Comparative transverse sections of (**A**) leaves, (**B**) stems, and (**C**) roots of *C. amblyocarpa* plant stained with Sudan III. Hand-made sections were stained with Sudan III for 5 min followed by washing with ethanol prior to microscopy examination. The lipid metabolites were only observed in the trichomes, particularly the heads of both leaves and stems, while in the idioblast cells of the root cortex.

Histochemistry staining of leaves, stems, and roots sections with Sudan III, a specific lipophilic stain, showed that the lipophilic metabolites were accumulated in the trichomes of both leaves and stems, particularly the heads of trichomes (Figure 5). On the other hand, the lipophilic metabolites in the roots were accumulated in idioblasts cells (Figure 5C).

The adapted mechanisms developed by *C. amblyocarpa* growing under drought conditions of UAE—in particular, the lipophilic metabolism—can be of importance to plant crops and farmers during drought conditions. Those lipophilic materials can provide crops growing in dry weather with an extra shield to protect them from water loss [59,60,61]. 

## 4. Conclusions

*C. amblyocarpa* growing in the UAE showed regular dicotyledonous characteristics with marked anatomical structures as a response to drought conditions. *C. amblyocarpa* growing in the arid desert of UAE demonstrated shaggy trichomes. The plants produced various lipid substances, including those produced for the purpose of detoxification, such as 2-naphthalene methanol, for pathogen protection, such as heneicosane, caryophyllene oxide, heptadecanal, tetratetracontane, spiro[4.5]decane, pyran, 2(1H)naphthalenone, and heptatriacotanol, and for drought tolerance mechanisms, such as hexatriacontane, tetracosane, 1-heptacosanol, phytol, n-nonadecanol-1, n-pentadecanol, octacosyl acetate, octadecanoic acid, and hexatriacontane. The protective mechanisms were more abundant in the low-water conditions and had more localization to the plant leaves, while drought tolerance mechanisms were more abundant in the roots. In regard to applications, such chemical ecology information can help to develop plant crops that can tolerate drought conditions and low-water availability. Identification of responsible genetic biomarkers of UAE *C. amblyocarpa* when compared to those from temperate regions will be of economical value in order to develop drought-tolerant plants. 

## Supplementary Materials

The following are available online at https://www.mdpi.com/2223-7747/8/5/132/s1, Figure S1: Chromatograms comparison for the plants at extra-water (black) and low-water (red) conditions. (**A**) Comparison of the plants roots. (**B**) Comparison of the plants stems. (**C**) Comparison of the plants leaves. (**D**) Comparison of the plants fruits, Table S1: Comparative GC-MS analysis of Cleome Amblyocarpa plant growing either in extra-water or low-water conditions of the arid desert of Sharjah, UAE. Metabolite average relative percentage of three replicates was displayed ± the standard error of the mean. The relative percentage of a metabolite represented in relation to total areas of all detected metabolites in an extract.
