# Supplementary material for: Lipophilic Metabolites and Anatomical Acclimatization of Cleome amblyocarpa in the Drought and Extra-Water Areas of the Arid Desert of UAE
Source: Plants (Basel). 2019 May 16;8(5):132. doi: 10.3390/plants8050132 (PMC6572330; doi:10.3390/plants8050132)
Supplement: Supplementary file 1 [file plants-08-00132-s001.pdf]

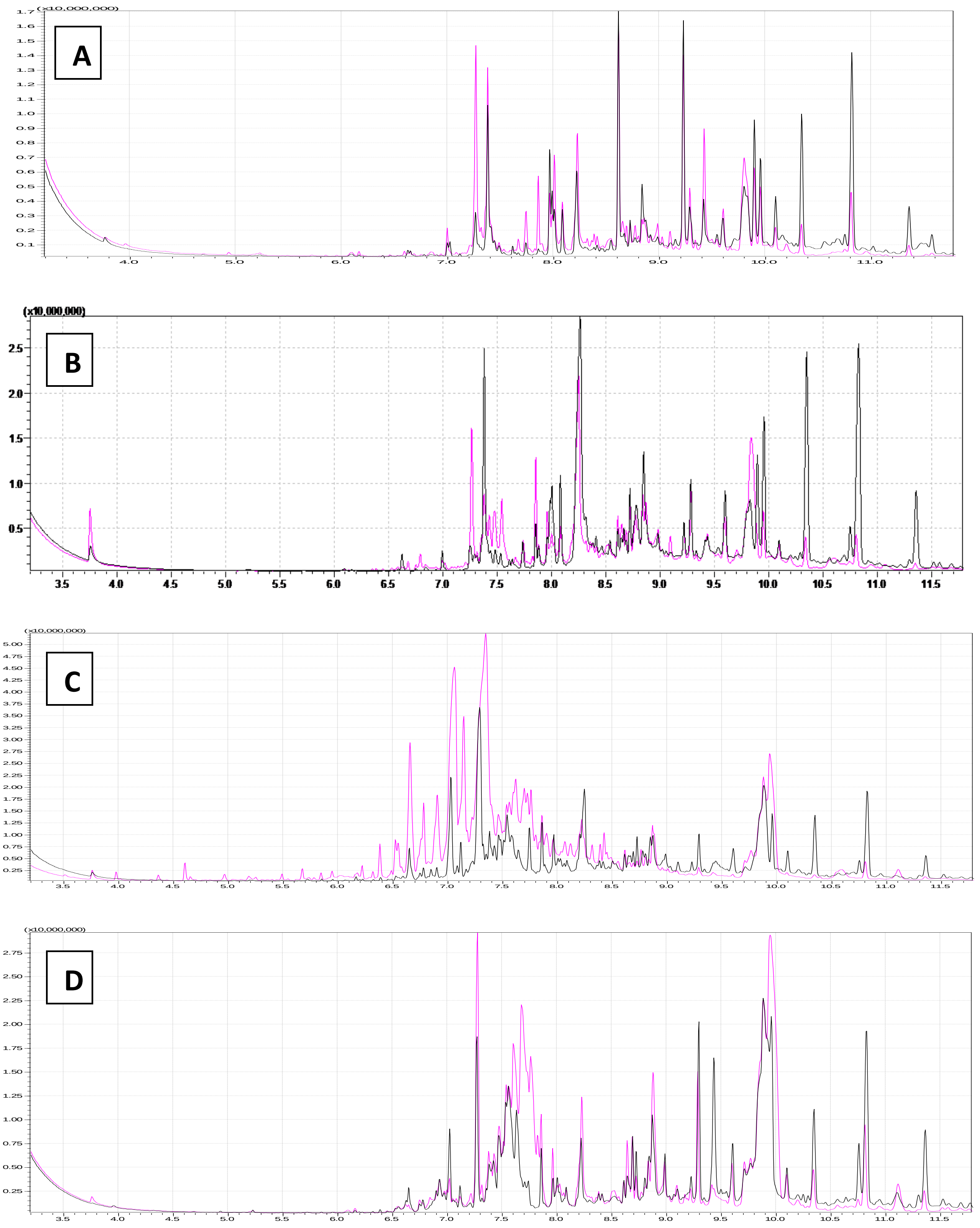

Supplementary Figure 1. Chromatograms comparison for the plants at extra-water (black) and low-water (red) conditions. (A) Comparison of the plants roots. **(B)** Comparison of the plants stems. **(C)** Comparison of the plants leaves. **(D)** Comparison of the plants fruits.

Supplementary Table 1. Comparative GC-MS analysis of Cleome Amblyocarpa plant growing either in extra-water or low-water conditions of the arid desert of Sharjah, UAE. Metabolite average relative percentage of three replicates was displayed ± the standard error of the mean. The relative percentage of a metabolite represented in relation to total areas of all detected metabolites in an extract.

Lipophilic Metabolites	Extra-Water Condition				Low-Water Condition			
	Roots	Stems	Leaves	Fruits	Roots	Stems	Leaves	Fruits
I- Tolerance								
Hexatriacontane	6.79 ± 0.6	nd	12.24 ± 0.7	26.51 ± 0.19	nd	12.05 ± 0.5	5.52 ± 0.25	40.98 ± 0.85
Tetracosane	nd	nd	nd	8.61 ± 0.35	nd	nd	nd	1.5 ± 0.09
1-Heptacosanol	9.56 ± 0.24	nd	nd	nd	2.83 ± 0.4	nd	nd	nd
n-Nonadecanol-1	17.05 ± 0.4	nd	nd	nd	12.54 ± 0.72	nd	nd	nd
n-Pentadecanol	6.08 ± 0.16	nd	nd	nd	nd	nd	nd	nd
Phytol	1.19 ± 0.16	1.48± 0.3	1.17 ± 0.06	1.07 ± 0.24	0.82 ± 0.06	1.65 ± 0.21	nd	0.55 ± 0.21
Tridecanal	nd	nd	nd	nd	nd	4.33 ± 0.18	nd	nd
9-Octadecenamide	nd	nd	nd	4.49 ± 0.17	5.18 ± 0.26	nd	nd	nd
Octacosyl acetate	nd	6.09 ± 0.27	nd	nd	nd	nd	nd	nd
Octadecanoic acid	5.3 ± 0.16	6.37 ± 0.14	nd	nd	nd	nd	nd	nd
Pentadecadien	nd	nd	nd	nd	nd	3.35 ± 0.27	nd	nd
II- Protective								
Heneicosane	3.38 ± 0.23	nd	nd	11.01 ± 0.26	nd	nd	nd	nd
Heptadecanal	7.32 ± 0.28	nd	nd	nd	9.71 ± 0.27	nd	nd	nd
Tetratetracontane	1.92 ± 0.2	nd	7.53 ± 0.64	nd	9.51 ± 0.31	nd	3.62 ± 0.06	1.26 ± 0.21
Caryophyllene oxide	nd	nd	4.65 ± 0.23	nd	nd	nd	13.85 ± 0.37	nd
Spiro[4.5]decane	nd	nd	nd	nd	nd	nd	5.41 ± 0.34	nd
2,6,10-Dodecatriene	nd	nd	nd	nd	nd	nd	2.97 ± 0.28	nd
Pyran	nd	nd	nd	nd	nd	3.32 ± 0.21	nd	nd
2(1H)Naphthalenone	nd	nd	nd	nd	nd	nd	3.85 ± 0.05	nd
1-Heptatriacotanol	nd	nd	nd	nd	nd	nd	5.81 ± 0.24	nd
Pentadecanoic acid	nd	nd	nd	nd	6.23 ± 0.44	nd	nd	nd
Naphthalene	nd	nd	1.15 ± 0.15	nd	nd	nd	3.71 ± 0.11	nd
III- Detoxification								
2-Naphthalenemethanol	nd	nd	10.73 ± 0.19	3.24 ± 0.7	9.56 ± 0.29	nd	22.4 ± 0.57	nd
IV- Growth and Development								
Squalene	2.37 ± 0.06	nd	nd	nd	nd	nd	nd	nd
Triacontanol (TRIA)	nd	nd	nd	nd	nd	nd	nd	7.4 ± 0.52
Triacontyl acetate	7.56 ± 0.24	nd	nd	nd	nd	nd	nd	nd
